# Prevalence of Anaemia, Deficiencies of Iron and Folic Acid and Their Determinants in Ethiopian Women

**DOI:** 10.3329/jhpn.v28i4.6042

**Published:** 2010-08

**Authors:** Jemal Haidar

**Affiliations:** School of Public Health, Addis Ababa University, PO Box 27285/1000, Addis Ababa, Ethiopia and Ethiopian Health and Nutrition Research Institute, PO Box 5654, Addis Ababa, Ethiopia

**Keywords:** Anaemia, Anaemia, Iron-deficiency, Community-based studies, Cross-sectional studies, Folic acid, Iron deficiency, Ethiopia

## Abstract

A cross-sectional community-based study with analytic component was conducted among Ethiopian women during June-July 2005 to assess the magnitude of anaemia and deficiencies of iron and folic acid and to compare the factors responsible for anaemia among anaemic and non-anaemic cases. In total, 970 women, aged 15-19 years, were selected systematically for haematological and other important parameters. The overall prevalence of anaemia, iron deficiency, iron-deficiency anaemia, deficiency of folic acid, and parasitic infestations was 30.4%, 50.1%, 18.1%, 31.3%, and 13.7% respectively. Women who had more children aged less than five years but above two years, open-field toilet habits, chronic illnesses, and having intestinal parasites were positively associated with anaemia. Women who had no formal education and who did not use contraceptives were negatively associated with anaemia. The major determinants identified for anaemia were chronic illnesses [adjusted odds ratio (AOR)=1.1, 95% confidence interval (CI) 1.15-1.55), deficiency of iron (AOR=0.4, 95% CI 0.35-0.64), and deficiency of folic acid (AOR=0.5, 95% CI 0.50-0.90). The odds for developing anaemia was 1.1 times more likely among women with chronic illnesses, 60% more likely in the iron-deficient and 40% more likely in the folic acid-deficient than their counterparts. One in every three women had anaemia and deficiency of folic acid while one in every two had iron deficiency, suggesting that deficiencies of both folic acid and iron constitute the major micronutrient deficiencies in Ethiopian women. The risk imposed by anaemia to the health of women ranging from impediment of daily activities and poor pregnancy outcome calls for effective public-health measures, such as improved nutrient supplementation, health education, and timely treatment of illnesses.

## INTRODUCTION

Anaemia remains a major public-health problem, affecting about a quarter of the world's population. Its adverse health consequences affect people with varied degrees of affluence and from all age-groups, particularly women of childbearing age and children (1). About two billion people are iron-deficient, with half of them manifesting clinical signs of anaemia (1,2). The micronutrient status of high-risk populations has recently received extensive attention as it adversely affects pregnancy outcome, working ability, intellectual capacity, and immunity, including enormous economic drainage (3–4).

Anaemia can result from non-nutritional factors, such as haemorrhage, infection, chronic disease states, or drug toxicity, and from nutritional ones, including deficiencies of iron, certain vitamins, copper, and protein (5–8). Iron deficiency remains the major cause of anaemia and is the most widespread single nutrient deficiency in the world. It is estimated that 75% of anaemia is related to iron deficiency, followed by folate and vitamin B12 deficiencies (9–11).

Identifying the magnitude of anaemia and its determinants in high-risk groups, such as women of childbearing age, would be essential for evidence-based intervention modalities, particularly in developing countries, such as Ethiopia, where the social conditions pose serious challenges to women (9). The nutritional status of women in Ethiopia, as in other developing countries, is low, and their daily workload is often enormous because of reproducing and ensuring the survival of their children (12). To improve the nutrition situation of Ethiopian women, there have been several interventions by the Ministry of Health through its Essential Nutrition Action (ENA) plan, comprising the supplementation of three major nutrients (vitamin A, iron, and iodine) and other promotive activities, such as exclusive breastfeeding, appropriate complementary feeding, and improved maternal and child nutrition (13).

Despite the efforts of the line ministry and its stakeholders, the recent demographic health survey report (EDHS) of 2005 showed that 27% of women, aged 15-49 years, were chronically malnourished, and about the same proportion suffered from anaemia with significant regional variations (14). Other nutritional data, such as iron status, however, were not documented in the EDHS 2005. The available information on the causes of iron-deficiency anaemia is limited in their capacity to be representative for the entire country, and some are misleading (15) and non-conclusive, despite the problem being among the 10 top morbidities (4). Although data on iron-deficiency anaemia are partial, the results show that iron-deficiency anaemia is a mild to moderate public-health problem in the country (16–18).

In 1998, for the first time, a relatively-representative sample of pregnant and lactating women was studied, and the prevalence rate of iron deficiency was 18.7% (16). Subsequent studies among 196 lactating women from urban slum communities of Addis Ababa, the capital city of the country, showed a prevalence of 22.3%, suggesting the problem to increase in its magnitude and reaching to a level of moderate public-health significance (17). As such, this study is a very important step forward to avail of evidence-based information on the magnitude of anaemia and compare the factors responsible for anaemia among anaemic and non-anaemic women to identify the potential causes of anaemia in apparently-healthy Ethiopian women.

## MATERIALS AND METHODS

### Study setting and design

A cross-sectional community-based study with analytic component was conducted during June-July 2005 in nine of the 11 regions of Ethiopia to assess the magnitude of anaemia, deficiencies of iron and folic acid and compare the factors responsible for anaemia among anaemic and non-anaemic women to identify the potential causes of anaemia in apparently healthy-looking Ethiopian women of childbearing age (15-49 years).

The criteria for determining the magnitude of anaemia recommended by the World Health Organization are based on the haemoglobin cut-off values for different ages and sex, with an additional epidemiological criterion for assessing the severity and magnitude of the problem in populations (19–22). When such data are not available, the prevalence of anaemia in high-risk group, such as women of childbearing age, could be used as a valid indication for the magnitude of the problem (23,24).

According to the 2004 Federal Ministry of Health of Ethiopia report, the total population is more than 73 million, more than 85% of whom are rural dwellers, depending on subsistent agricultural economy. The staple crops are *teff* (*Eragrostis tef*) and cereals in the north and central parts; *enset* (*Ensete ventricosum*), cassava (*Manihot esculenta*), maize (*Zea mays*), cereals, and root crops in the South and Southwest; and sorghum and maize in the East of the country (25).

Of the 11 regional states in the country, namely Tigray, Afar, Amhara, Oromiya, South Nation and Nationalities People, Benishangul-Gumuz, Harari, Diredawa, Addis Ababa Gambella, and Somali, nine were included in the study because Gambella and Somali regions were inaccessible at the time of the study for security reason. Altogether, 270 clustered villages across the nine regions were randomly selected. In each selected cluster (a cluster is equivalent to the smallest administrative unit commonly known as *kebele*), all women of childbearing age were invited and those who agreed were considered.

Multi-stage cluster-sampling methods were applied to select the subjects. In each regional state, cumulative populations were calculated, and the attributed number was assigned. The sampling interval was then calculated by dividing the total number of the study subjects with the number of clusters. A random number was drawn using a random number table. The first cluster was selected based on this number table. To select the remaining clusters, the sampling interval was added sequentially to the random number until all the 270 clusters were selected.

In each cluster, households were selected by locating the centre of the village/*kebele* and spinning a pen and proceeding to the direction that the pen pointed. Household along the direction to the boundary of the locality was counted and numbered. A random number was picked from a random table to select the first house to examine and interview eligible women. Then the subsequent households in the identified direction were visited until a sample size of 100 women was reached (assuming 18.7% prevalence of anaemia with worst acceptable of 4.5% and 99% confidence level with a design effect of 2). In total, 27,000 apparently-healthy women were enrolled using probability proportional to the population size for the detection of pallor. While for biological sample collections and administrations of the questionnaire, 970 (5%) women were selected systematically and interviewed. Those women who were not present at the time of the survey were revisited and examined clinically for pallor.

### Collection of data

Before the survey, two doctors, two nurses, and two laboratory technicians were recruited from each respective region and trained for one week to serve as data collectors by the principal investigator in Nazareth town, 99 km east of the capital. The training was focused on how to perform standardized clinical examination of pallor and on interviewing techniques of the structured questionnaire. The result of each examiner was compiled, and the variation was assessed. The standardization procedure continued until the inter-observer variation was negligible. The doctors conducted the clinical examination while the nurses interviewed the sampled households.

The questionnaire used in the present study was composed of sociodemographic information (age, education, marital status, family-size, number of children aged less than five years (under-five children); obstetric history (pregnancy, parity, birth-spacing, and family-planning method-use); illnesses (malaria and any febrile illnesses in the last two weeks and others); intestinal parasite insestation (hookworm, ascariasis, schistosomiasis, trichuriasis, and ameobiasis); and qualitative dietary information, such as how frequently foods from plant and animal sources were consumed weekly and yearly respectively other than the staple diet of the sampled households.

To get a rough estimate of dietary intake, a simplified food-frequency questionnaire, slightly modified to the local circumstances, from the Helen Keller International Food Frequency Questionnaire was used, in addition to the staple food intake (26). For the preparation of the list, a preliminary market survey and interview with elders on the type of food items produced locally and available in the market were conducted. Twenty food items available and known to have vitamin A activity, iron, high fat, and protein content were prepared. The plant sources included banana, bean, bread, broccoli, cabbage, cassava leaves, groundnut, morinaga, orange, peanut, potato, rice, spinach, and Swiss chard whereas the animal sources of food comprised meat sources: beef, egg, fish, liver, milk, poultry, and an ‘other’ option. Responses were grouped according to the frequency of consumption of meat and vegetables.

### Collection of samples

Trained laboratory technicians aseptically collected all biological samples which were analyzed partly at the spot. Stool samples were collected and examined for ova and parasites microscopically as described by Kruger *et al*. (27) at the spot, and haemoglobin was analyzed using the portable hemoglobinometer HemoCue (HemoCue AB, Ängelholm, Sweden) methods at the study sites. Anaemia was then defined as Hgb <11 g/dL in pregnant women and <12 g/dL for non-pregnant women after an adjustment was made for pregnancy and altitude at the field based on the recommendations of the International Nutritional Anemia Consultative Group (28).

Registered laboratory technicians drew a sample of 5 mL fasting venous blood from each subject into a sterile tube containing no anti-coagulant. Blood samples were centrifuged and stored frozen at-20°C and transported to the Ethiopian Health and Nutrition Research Institute (EHNRI) where it was analyzed for serum ferritin using an enzyme-linked immunosorbent assay with a fully-automated Elecsys 1020 using commercial kits purchased from Boerrhinger Maneheim, Germany at the EHNRI. Severe and moderate iron deficiency was considered when serum ferritin was below 12 and 50 μg/L respectively to balance the effect of infection as recommended by the WHO for developing countries (29). The same instrument was used for measuring serum folate using commercial kits purchased from the same company at the EHNRI with its quality-control material purchased from the Roche Company to ascertain the quality of the tests.

Controls for various concentration measurements were based on fully-automated Elecsys 1020 analyzer enzyme linked-immunosorbent assays technique and were run as a single determination at least once every 24 hours when the test is in use, once per repeated kit, and after every calibration. Values obtained fall within the defined limits. By virtue of being simple, concentration of serum folate was used for estimating the magnitude of current status of folate, and accordingly, values below 3 and 6 ng/mL were taken as indicative of severe and marginal folate deficiency respectively while the value above 20 ng/mL was considered to be excess (30,31).

### Analysis of data

The SPSS software (version 12) was used for data-entry and cleaning, and all the detected inconsistencies were addressed and outliers were dropped. Standard tabulations were also generated in which the outliers were identified before subjecting the data to analysis. The chi-square test was performed to determine the differentials of anaemia by explanatory variables. Pearson's chi-square test of independence was performed to determine the existence of significant association of sociodemographic and other important variables with anaemia. Stepwise backward logistic regression model was applied to test the observed significant variables found in the chi-square test while controlling the confounding effect of other variables. A p value of <0.05 denoted the significance in differences.

### Ethics

The Research and Ethical Clearance Committee of the EHNRI approved the study. An informed written consent was obtained from all subjects for their participation after the nature of the study was fully explained to them in their local languages by thumbprint or signature in the consent form. At the end of collections of biological samples, all the subjects found to be positive for intestinal and haemo-parasites were treated free of charge at the expense of the project at the spot. Likewise, nine women with severe anaemia detected in the process were also treated at the spot with full course of medicinal iron tablet containing folic acid based on the national micronutrient guideline.

## RESULTS

Of the 27,000 women recruited, only 22,861 (86.7%) were considered for the clinical assessment for the detection of pallor (data not shown).

To identify the potential determinants of anaemia, only those (n=970; 84.8%) who had complete haematological and the important parameters are presented in the subsequent sections.

Table 1 shows that the majority (58.8%) of the women had formal education, most (82.2%) were aged 25-35 years, and 60.5% were married. A few (9.7%) were pregnant. The proportion of women with family-size greater than five was 58.5%. The proportions of women with more than two children, narrow birth-spacing, who used no familyplanning methods, had illnesses and intestinal parasites, consumed foods from plant and animal sources less frequently were 13.1%, 74.0%, 46.3%, 13.0%, 13.7%, 41.5%, and 43.5% respectively. The prevalence of anaemia, iron deficiency, iron-deficiency anaemia, and deficiency of folic acid was also 30.4%, 50.1%, 18.1%, and 31.3% respectively.

**Table 1.** Distribution of selected characteristics of Ethiopian women aged 15-49 years

Selected characteristics	No.	%
Formal education		
No	570	58.8
Yes	400	41.2
Age (years)		
15-24	249	25.7
25-35	587	60.5
36-49	134	13.8
Marital status		
Marital union	797	82.2
Non-marital union	173	17.8
Pregnant		
No	876	90.3
Yes	94	9.7
Family size		
>5	58.5	567
1-5	403	41.5
Number of children aged less than 5 years		
1-2	843	86.9
>2	127	13.1
Toilet-use		
Latrine	488	50.3
Open field	482	49.7
Birth-spacing (years)		
>2	252	26.0
1-2	718	74.0
Family-planning method-use		
No	449	46.3
Yes	521	53.7
Illness before 2 weeks		
No	844	87.0
Yes	126	13.0
Intestinal parasite		
Yes	133	13.7
No	837	86.3
Consumption of foods from animal sources (yearly)		
>3 times	567	58.5
≤3 times	403	41.5
Consumption of foods from plant sources (weekly)		
>3 times	548	56.5
≤3 times	422	43.5
Consumption of tea		
No	216	22.3
Yes	754	77.7
Haemoglobin		
Anaemic	295	30.4
Non-anaemic	675	69.6
Serum ferritin		
<50 μg/L (iron-deficient)	486	50.1
≥50 μg/L (iron-sufficient)	484	49.9
Iron-deficiency anaemia		
Low Hb* + SF <50 μg/L	176	18.1
Non-anaemic + SF >50 μg/L	794	81.9
Serum folic acid		
<6 ng/mL (FAD)	304	31.3
≥6 ng/mL (sufficient)	666	68.7

*Haemoglobin <11 g/dL in pregnant women and <12 g/dL in non-pregnant women;

FAD=Folic acid-deficient;

SF=Serum ferritin

The most frequently-encountered intestinal helminthes were *Ascaris lumbricoids* (35.3%; 47/133), followed by *Trichuris trichiura* (28.6%; 38/133), *Entamoeba histolytica* (22.6%; 30/133), *Schistosoma mansoni* (19.5%; 26/133), and hookworm or *Ancylostoma duodenale* (16.5%; 22/133) (Fig.).

**Fig.**
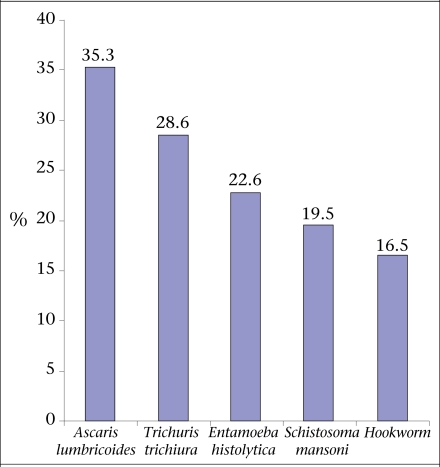
Prevalence of intestinal parasites among Ethiopian women aged 15-49 years

Percentages exceed 100% because of multiple infections

Although the prevalence of anaemia was slightly higher among women with no formal education (31.9%), relatively older women (36.6%), married women (30.7%), pregnant women (30.5%), family-size of >5 (31.0%), mothers with more than two children (34.6%), narrow birth-spacing (31.6%), who used no family-planning methods (37.4%), and harboured no intestinal parasites (28.6%), the differences were not significant. Although not significant, the prevalence of anaemia was also slightly higher among women who had consumed tea (30.2%) and had less frequently eaten foods from animal (32.0%) and plant (31.8%) sources than their counterparts. The occurrence of anaemia was significantly higher among women who had a history of illnesses (71.6%) and deficiencies of iron (37.0%) and folic acid (24.5%) (Table 2).

**Table 2.** Selected sociodemographic, health and dietary factors associated with prevalence of anaemia among Ethiopian women aged 15-49 years

Variable	Total	Anaemia		χ^2^	p value
		No.	%		
Formal education					
No	570	182	31.9		
Yes	400	113	28.3	0.2	0.1
Age (years)					
15-24	249	78	31.9	3.3	0.1
25-35	587	168	28.6		
36-49	134	49	36.6		
Marital status					
Marital union	797	245	30.7	0.7	0.3
Non-marital union	173	50	28.9		
Pregnant					
No	876	267	30.5		
Yes	94	28	29.8	0.5	0.3
Family-size					
>5	567	176	31.0		
1-5	403	119	29.5	0.2	0.3
Number of children aged less than 5 years					
1-2	843	251	29.8		
>2	127	44	34.6	1.2	0.1
Toilet-use					
Latrine	488	147	30.1		
Open field	482	148	30.7	0.04	0.4
Birth-spacing (years)					
>2	252	68	27.0
1-2	718	227	31.6	1.8	0.9
Family-planning method-use					
No	449	168	37.4		
Yes	521	127	24.3	1.7	0.1
Illness before 2 weeks					
None	844	240	28.4		
Pneumonia	39	14	35.9		
Malaria	42	16	38.1	16.7	0.001
Chronic illness (TB)	45	25	55.6		
Intestinal parasite					
Yes	163	38	28.6		
No	807	257	30.7	0.2	0.3
Consumption of foods from animal sources (yearly)					
>3 times	548	161	29.43		
≤3 times	422	134	31.75	0.6	0.2
Consumption of foods from plant sources (weekly)					
>3 times	567	166	29.3		
≤3 times	403	129	32.0	0.8	0.2
Consumption of tea					
No	216	67	21.0		0.4
Yes	754	228	30.2	0.04
Serum folic acid					
<6 ng/mL (FAD)	666	163	24.5		
≥6 ng/mL (sufficient)	304	132	43.4	35.4	0.001
Serum ferritin					
<50 μg/L (iron-deficient)	481	118	37.0		
≥50 μg/L (iron-sufficient)	489	177	23.9	19.6	0.001

FAD=Folic acid deficiency;

TB=Tuberculosis

To see the effect of parasitic infestation by types of anaemia, chi-square test was run. Interestingly, the occurrence of all types of anaemia was lower among women who had no parasites, suggesting that intestinal parasite in the present study was less likely to be the causative agent of anaemia (Table 3).

**Table 3.** Associations of all types of anaemia with prevalence of intestinal parasite among Ethiopian women aged 15-49 years

Characteristics	Total	Have intestinal parasite		χ^2^	p value
		No.	%		
Haemoglobin (Hb)					
Anaemic*	295	38	3.9		
Non-anaemic	675	95	9.8	0.2	0.6
Serum ferritin					
<50 μg/L (iron-deficient)	481	51	5.2		
≥50 μg/L(iron-sufficient)	489	63	6.5	2.6	0.1
Iron-deficiency anaemia					
Anaemic + SF <50 μg/L	176	23	2.4		
Non-anaemic + SF >50 μg/L	794	110	11.3	0.08	0.7
Serum folic acid					
<6 ng/mL (FAD)	304	33	3.4		
≥6 ng/mL (FA sufficient)	666	100	10.3	3.05	0.08

*Anaemic refers to Hb <11 g/dL in pregnant and <12 g/dL in non-pregnant women;

FA=Folic acid;

FAD=Folic acid deficiency (serum folic acid deficiency <6 ng/mL);

ID=Iron deficiency (serum ferritin <50 ug/L);

IDA=Iron-deficiency anemia (anaemia + serum ferritin <50 μg/L);

SF=Serum ferritin

To control the effect and predict the most important determinants of anaemia, a stepwise logistic regression analysis was performed. In the model, those independent variables that had marginal and significant associations in the chi-square tests were fed to the regression models. Women having children above two, using open field as toilet, suffered from chronic illnesses, and had intestinal parasites were positively associated with anaemia. Women with no formal education and who did not use contraceptives were negatively associated with anaemia. The major determinants identified for anaemia were chronic illnesses [adjusted odds ratio (AOR)=1.1, 95% confidence interval (CI) 1.15-1.55) and deficiencies of iron (AOR=0.4, 95% CI 0.35-0.64), and folic acid (AOR=0.5, 95% CI 0.50-0.90). The odds for developing anaemia was 1.1 times more likely among women with chronic illnesses, 60% more likely in the iron-deficient, and 48% more likely in the folic acid-deficient than their counterparts.

## DISCUSSION

Overall, the evidence presented denotes that one in every five women had iron-deficiency anaemia, one in every three women had anaemia, and one in every two had iron deficiency and qualifying for moderate public-health significance based on the WHO cut-off point set for haemoglobin (23,24). In this study, nearly 37% (n=118) of the cases with anaemia were associated with low ferritin values. It may be that, even when the cut-off values of haemoglobin were adjusted for physiological status of the women, the prevalence of anaemia was underestimated; probably, a certain proportion of women might have had a subclinical infection that raised ferritin values. Therefore, women who had low haemoglobin and normal ferritin values were considered as having anaemia from causes other than iron deficiency, such as deficiencies of folic acid and vitamin A and B1_12_, although the latter two were not measured (32). However, low serum folic acid was prevalent among one in every three women.

More than one-third (n=118) of the anaemic women had low ferritin levels, and nearly a quarter (n=153) of the anaemic women had deficiency of folic acid, suggesting that anaemia was related to micronutrient deficiencies. The prevalence of 18.1% of iron-deficiency anaemia found in this study is similar to the findings of a previous study (16). The concordance of the relatively recent estimates coupled with an overall rate of anaemia from deficiency of folic acid and other causes of 30.6% found in this study denote deprivation of iron and folic acid from their diet and merits greater consideration.

Another interesting comparison was found when contrasting various maternal characteristics with and without anaemia. Over half of the anaemic women observed were those who had a history of illnesses, suggesting that infection was an important cause, and the differences noted were significant. This is explained by the fact that infection causes anaemia through loss of nutrients, decreasing appetite, decreasing efficiency of absorption, and use of nutrients (33). Although it would be expected to find a higher prevalence of anaemia among women with parasitic infestations due to the same line of reasoning forwarded for infection, this was not the case probably due to the types of parasitic infestation harboured by the women at the time of the study as nearly three-fourths of them had the less-invasive intestinal parasites, such as ascariasis and trichuriasis. This finding suggests that nutrient deficiencies and illnesses rather than parasitic infestations are primarily the causes of anaemia in Ethiopian women.

The occurrence of anaemia being more prevalent in the present study among the older age-group was probably because of the high fertility rate and greater loss of menstrual blood. As expected, due to cumulative obstetric conditions of women, the prevalence of all types of anaemia was also slightly higher among pregnant women and mothers who had two or more children with narrow birth-spacing than their counterparts, which concurs with previous pocket-studies done in the country (13,16). This could be attributed to the increased requirements for iron and folic acid during pregnancy and pregnancy-related maternal exhaustions. Such an association of anaemia with age, education level, physiological status, hygiene practices, infection, and parasitic infestation is not uncommon in most countries, which needs attention for early actions by public healthcare services (34,35).

**Table 4.** Predictors of anaemia in Ethiopian women aged 15-49 years

Characteristics	Total	Anaemia	Adjusted odds ratio (95% CI)
Formal education				
Yes	400	64 (16.0)	1
No	570	182 (31.9)	0.86 (0.6-2-1.17)
Number of children aged less than 5 years				
1-2	843	251 (29.8)	1
>2	127	44 (34.6)	1.3 (0.81-1.9)
Toilet-use				
Latrine	488	147 (30.1)	1
Open field	482	148 (30.7)	1.1 (0.88-1.60)
Family-planning method-use				
Yes	449	168 (32.3)	1
No	521	127 (28.3)	0.9 (0.60-1.26)
Illness before 2 weeks				
None	844	14 (35.9)	1
Pneumonia	39	16 (38.1)	0.6 (0.30-1.22)
Malaria	42	25 (55.6)	0.6 (0.32-1.21)
Chronic illness (TB)	45	240 (28.4)	1.1 (1.15-1.55)*
Intestinal parasite				
Yes	163	38 (23.3)	1
No	807	257 (31.8)	1.1(0.74-1.6)
Serum ferritin				
≥50 μg/L (normal)	489	177 (23.9)	1
<50 μg/L (iron-deficient)	481	118 (37.0)	0.4 2(0.35-0. 64)*
Serum folic acid				
≥6 ng/mL (normal)	304	132 (43.4)	1
<6 ng/mL (FAD)	666	163 (24.5)	0.52 (0.50-0.90)*

*p=0.001;

Results from multivariate analysis—adjusted for formal education, number of children aged less than five years, toilet-use, family-planning use, illness before 2 weeks, intestinal parasite, serum folic acid, and serum ferritin;

FAD=Folic acid-deficient;

TB=Tuberculosis;

Figures in parentheses in column 3 indicate percentages

Several studies have demonstrated a positive relationship between both qualitative and quantitative adequacy of the diet (36). Although not statistically significant, anaemia was slightly higher among women who had consumed foods from both animal and plant sources less frequently than their counterparts. Such a weak association noted could be partly attributed to the cultural food taboos and religious fasts persisting among different ethnic and gender groups. Taboos have also limited the development and use of certain plant and animal food resources, with detrimental effect particularly on mothers. Avoidance of food in the form of reluctance to eat vegetables and fruits is also widespread in some areas (37). Although the practice of vegetable consumption was relatively better than food from animal sources, its consumption is still low, and it is consumed by less than half of the subjects, which again underlines the need for intensification of backyard gardening, along with nutrition education to ensure availability and increased consumption of vegetables and fruits.

### Limitations

Although the present study had a large sample-size with a good response rate, there is still a possibility of biases because of the exclusion of most at-risk regions, such as the pastoralists, which might have underestimated the prevalence rates of all types of anaemia. The dietary data were based on qualitative information and, thus, could not estimate the precise assessment of the nutrient intake. Additionally, the absence of C-reactive protein assessment was another limitation, which would have ruled out the presence of infection and, thus, validate the actual level of iron deficiency in the women studied. The result presented for folic acid could also be underestimated because erythrocyte folic acid concentration was not assessed which might have an added value in the assessment. Likewise, vitamin B_12_ was also not measured due to resource constraints.

### Conclusions

The present study revealed an overall prevalence of anaemia which is qualifying for moderate public-health significance based on the WHO standards (22). The study has also demonstrated that illnesses and deficiencies of iron and folic acid were some identified explanatory variables of anaemia. The observed deficiencies of iron and folic acid suggest the presence of deficiencies of multiple micronutrients. Although the contribution of parasitic infestation was not significant, its potential effect in causing anaemia should not be neglected. Iron and folic acid were the principal micronutrient deficiencies in Ethiopian women. Anaemia associated with illnesses (71.6%) was more prevalent than with deficiencies in iron (34%) and folic acid (24%).

The fact that deficiencies of multiple micronutrients are demonstrated in this study, there is a need to establish effective preventive public-health nutrition programmes to address the problem of anaemia in the country. Thus, the ongoing control and prevention of micronutrient-deficiency strategy needs to reach all the vulnerable women to minimize and avert the negative health consequences ranging from impediment of daily activities and poor pregnancy outcome (38). The strategies consist of supplementation of iron and folic acid during the pregnancy and lactation period; treatment of helminthes and illnesses; sleeping under mosquito net; nutrition education; and improved sanitation.

## ACKNOWLEDGEMENTS

This study would not have been possible without funding support of the Ethiopian Federal Ministry of Health and Ethiopian Health and Nutrition Research Institute. The author thank**s** all the field supervisors and senior medical technologist of the Institute for their valuable support during the analysis of serum. The author also thank**s** the doctors, nurses, and laboratory technicians of the respective regions who served as data-collectors. The support by the respective region, including the communities, is also highly acknowledged.
